# Semiquantitative assessment of ^99m^Tc-MIBI uptake in parathyroids of secondary hyperparathyroidism patients with chronic renal failure

**DOI:** 10.3389/fendo.2022.915279

**Published:** 2022-09-08

**Authors:** Dafu Yu, Lin Zou, Yao Jin, Mingxiang Wei, Xiaoqun Wu, Lingjing Zuo, Mingkang Wu, Yong Jiang

**Affiliations:** ^1^ Department of Nuclear Medicine, The Eighth Affiliated Hospital, Sun Yat-Sen University, Shenzhen, China; ^2^ Department of Nuclear Medicine, The First People’s Hospital of Yunnan Province, Kunming, China; ^3^ Department of Laboratory Medicine, The First Affiliated Hospital of Chongqing Medical University, Chongqing, China; ^4^ Department of Respiratory Disease, The First People’s Hospital of Yunnan Province, Kunming, China

**Keywords:** secondary hyperparathyroidism, semiquantitative assessment, chronic renal failure, parathyroids, ^99m^Tc-methoxyisobutylisonitrile (MIBI)

## Abstract

**Objective:**

To explore the valuably influential factors and improve the diagnostic accuracy and efficiency of ^99m^Tc-methoxyisobutylisonitrile (MIBI) uptake in parathyroids of secondary hyperparathyroidism (SHPT) patients with chronic renal failure (CRF).

**Methods:**

The correlation analysis was performed between clinical indices related to CRF and ^99m^Tc-MIBI uptake intensity TBR (the gray value mean ratio between the parathyroid target and the bilateral neck background, semiquantitatively calculated with ImageJ software). All clinical indices and TBRs were compared by a three- or two-level grouping method of MIBI uptake, which was visually qualitatively assessed. The three-level grouping method comprised slight, medium, and high groups with little, faint, and distinct MIBI concentration in parathyroids, respectively. The two-level grouping method comprised insignificant and significant groups with TBR greater than or less than 0.49–0.71, respectively.

**Results:**

MIBI uptake was significantly positively related to patient age, CRF course, hemodialysis vintage, serum parathyroid hormone (PTH), and alkaline phosphatase (AKP) but was significantly negatively related to serum uric acid (UA). MIBI washout was significantly positively related to patient age but was significantly negatively related to serum phosphorus (P) and calcium (Ca) × P. Oral administration of calcitriol and calcium could significantly reduce the MIBI uptake. MIBI uptake tendency might alter. Such seven indices, namely the MIBI uptake, CRF course, hemodialysis vintage, serum AKP, calcium, cysteine proteinase inhibitor C, and PTH, were comparable between the slight and medium groups but were significantly different between the slight and high groups or between the medium and high groups. The above seven indices plus blood urea nitrogen/creatinine were all significantly different between the insignificant and significant groups. All above significances were with *P* < 0.05.

**Conclusions:**

Patient age, CRF course, hemodialysis vintage, serum PTH, AKP, UA, phosphorus, Ca × P, oral administration of calcitriol and calcium, and parathyroids themselves can significantly influence MIBI uptake in parathyroids of SHPT patients with CRF. The two-level grouping method of MIBI intensity should be adopted to qualitatively diagnose the MIBI uptake.

## Introduction

Secondary hyperparathyroidism (SHPT) is a common complication in patients with chronic renal failure (CRF). Parathyroidectomy (PTx) was suggested by the National Kidney Foundation Kidney Disease Outcomes Quality Initiative (KDOQI) to treat severe SHPT when drug regime failed (1). PTx is the preferred treatment (2) and the only cure (3) for severe drug-resistant SHPT.

Precise identification of the diseased parathyroid lobe(s) is the prerequisite for surgical operation (4). ^99m^Tc-methoxyisobutylisonitrile (MIBI) scintigraphy has many advantages (5, 6) in diagnosing, differentiating, and locating diseased parathyroid(s). Different from the fact that the patients with primary hyperparathyroidism generally have only one lesion (7), patients with SHPT usually have several lesions including ectopic glands (6) and multiglandular disease (5, 8). Some SHPT patients with parathyroid hyperplasia or adenoma show false negativity (5). These complex characteristics increase the accuracy limitation of ^99m^Tc-MIBI scintigraphy in positioning the parathyroid(s) relative to SHPT (3, 5, 9, 10).

In diseased parathyroids, significant ^99m^Tc-MIBI accumulation foci can evidently demonstrate the location of being planned surgical resection, but how should those insignificant ^99m^Tc-MIBI accumulation foci be dealt with? If they are considered negative foci, in fact, their serum parathyroid hormone (PTH) is higher than the normal range; if they are considered positive foci, the location and numbers of diseased parathyroid(s) cannot be confirmed because of the low spatial resolution of planar imaging and indistinct boundary from surrounding tissues.

Herein, semiquantitative assessment of the ^99m^Tc-MIBI accumulation level in parathyroid lobes was utilized to analyze the functional status of MIBI uptake. Basing on these semiquantitative assessment results, the analysis of the relativity of ^99m^Tc-MIBI accumulation to several factors (11) was performed, which might help to understand the characteristics of insignificant ^99m^Tc-MIBI accumulation foci.

## Materials and methods

### Patients

Retrospective investigation was performed in 151 SHPT inpatients (75 women, 76 men) from December 2015 to January 2021. These patients were with CRF course 48.34 ± 51.76 (0–223) months [(mean ± SD) (minimum – maximum value); the latter expressions were the same as this one], age 47.91 ± 14.95 (14–78) years, and hemodialysis vintage 26.83 ± 44.47 (0–223) months. CRF course was confirmed from the day on which the patients were firstly diagnosed with chronic renal failure to the day on which ^99m^Tc-MIBI scintigraphy was performed. All of them had high serum PTH levels of 1379.10 ± 765.75 (276–4620) (normal range 15–68.3) pg/mL and parathyroid MIBI scintigraphy. Most of them had measured serum ionized calcium (Ca), phosphorus (P), alkaline phosphatase (AKP), blood urea nitrogen (BUN), uric acid (UA), creatinine (Cre), cysteine proteinase inhibitor C (CPI), calcitonine, ferritin (Fer), vitamin B12 (VitB12), folate, erythropoietin (EPO), hemoglobin (Hb) (**Table 1**), and thyroid function on an empty stomach. All above biochemical tests were completed 2 days before every hemodialysis cycle. Patients were excluded from the analysis based on the following criteria: history of previous parathyroid or thyroid surgery, abnormal MIBI accumulation beyond thyroid contour (for decreasing the possibility of MIBI concentration in non-parathyroid tissue because only little patients had undergone parathyroidal surgery), or primary hyperparathyroidism. The patients’ inclusion criteria were as follows: serum PTH > 207 pg/mL, disease history of being confirmed CRF, and dual-phase MIBI scintigraphy with the time difference < 5 days between laboratory biochemical tests and MIBI imaging.

**Table 1:** The relativity of some indices to ^99m^Tc-MIBI uptake TBRs.

Indices (unit)	Normal range	Mean	Cases	AvgE	MinMeanE	AvgD	MinMeanD	MinWash	MaxWash
Age (years)		47.91	151	**-0.290^c^**	**-0.309^c^**	**-0.158^a^**	**-0.221^b^**	-0.044	**0.181^a^**
Course (months)		48.34	151	**-0.181^a^**	**-0.267^c^**	**-0.181^a^**	**-0.239^b^**	-0.083	0.030
Hemodialysis months		26.83	151	-0.135	**-0.249^b^**	**-0.195^a^**	**-0.296^c^**	-0.128	-0.017
GFR (mL/min)	> 80	18.79	37	-0.091	0.082	-0.286	-0.245	-0.203	-0.139
AKP (U/L)	35 -104	184.23	151	-0.134	**-0.224^b^**	-0.101	**-0.185^a^**	0.007	0.110
BUN (mmol/L)	2.0 - 7.1	27.97	151	0.043	0.068	-0.015	0.051	-0.070	-0.053
Creatinine (μmol/L)	45 - 84	879.95	151	0.020	0.010	-0.099	-0.050	-0.074	-0.083
UA (μmol/L)	178 - 416	498.65	151	0.127	0.017	**0.188^a^**	**0.215^b^**	0.065	0.013
BUN/Creatinine		33.97	151	0.045	0.079	0.095	0.109	-0.002	0.018
Ca (mmol/L)	2.1 - 2.6	2.15	151	-0.107	-0.157	-0.026	-0.112	0.041	0.061
Phosphorus (mmol/L)	0.8 - 1.45	1.96	151	0.078	0.078	-0.127	-0.074	**-0.210^b^**	**-0.166^a^**
Ca × P		4.19	151	0.014	-0.018	-0.119	-0.118	**-0.166^a^**	-0.111
CPI (mg/L)	0.41 - 0.98	6.75	151	-0.082	-0.145	-0.060	-0.095	0.035	0.010
Calcitonine (pg/mL)	< 2	6.30	151	0.083	0.033	-0.003	0.003	-0.029	-0.072
PTH (pg/mL)	15 - 68.3	1379.10	151	-0.045	-0.177	-0.077	**-0.183^a^**	-0.072	0.050
Ferritin (ng/mL)	4.63 - 204	398.19	151	-0.078	-0.122	-0.006	-0.017	0.032	0.079
VitB12 (pmol/L)	138 - 652	453.85	151	-0.025	-0.035	-0.118	-0.107	**-0.160^a^**	-0.079
Folate (nmol/L)	7 - 46.4	22.99	151	-0.008	-0.009	-0.107	-0.098	-0.064	-0.089
EPO (mIU/L)	3.7 - 29.5	37.61	151	0.022	-0.008	-0.013	-0.046	0.004	-0.021
Hb (g/L)	110 - 150	93.20	151	-0.100	-0.090	-0.130	-0.141	-0.086	-0.013

AvgE and AvgD, the average value of four TBRs in parathyroid ROIs of each patient during early and delayed phase, respectively; MinMeanE and MinMeanD, the minimum one among four TBRs in parathyroid ROIs of each patient during early and delayed phase, respectively; MinWash and MaxWash, the minimum and maximum one among four parathyroid washout ratios, respectively; GFR, glomerular filtration rate; AKP, alkaline phosphatase; BUN, blood urea nitrogen; Ca, serum calcium ion; UA, uric acid; CPI, cysteine proteinase inhibitor C; PTH, parathyroid hormone; VitB12, vitamin B12; EPO, erythropoietin; Hb, hemoglobin; r, Pearson’s relativity; P, two-tailed P value. Pearson’s correlation was performed for analysis. ^a^P < 0.05. ^b^P < 0.01. ^c^P < 0.001. **Bold values** show significance after statistical analysis.

### Control group

A total of 40 subjects (21 women, 19 men) from December 2015 to January 2021 were retrospectively investigated as the control group. These subjects were of age 47.73 ± 15.07 (15–84) years old, with serum AKP 76.71 ± 20.08 (45–118) U/L, BUN 5.27 ± 1.63 (2.9–10.2) mmol/L, Cre 67.77 ± 18.70 (30–107) μmol/L, BUN/Cre 79.65 ± 36.25 (13.81–229.4), UA 345.76 ± 104.57 (151–603) μmol/L, Ca 2.27 ± 0.14 (2.02–2.66) mmol/L, P 1.01 ± 0.19 (0.49–1.36) nmol/L, Ca × P 2.27 ± 0.65 (1.30–3.07), PTH 83.92 ± 23.33 (21–123.70) pg/mL, Hb 142.13 ± 19.91 (74–175) g/L, normal thyroid function, and serum calcitonine. The control group without history of chronic renal failure comprised eight normal subjects (with normal serum PTH, calcium, and phosphorus) and 32 mild idiopathic osteoporosis patients (with serum PTH 70.0–123.7 pg/mL, normal serum calcium and phosphorus, and mildly low bone density). All of them underwent ultrasound and/or CT check to exclude the subjects with abnormal nodules or nodes in thyroid contours and around necks. All of them had not measured serum CPI, ferritin, VitB12, folate, EPO, and 25-OH VitD but had planar dual-phase MIBI scintigraphy. All control subjects did not take antiresorptive medication, calcium, calcidiol, or calcitriol supplements.

### Imaging study protocol

All patients received an intravenous injection of 740 MBq ^99m^Tc-MIBI. Early and delayed scans from the lower part of the head to the upper thorax were performed 20 min and 2 h post injection, respectively. All these scans were performed in the supine and neck-extended positions in the anterior–posterior view. The imaging acquisition used Philips Precedence SPECT/CT 6 (Philips Medical Systems, Netherlands) with a matrix of 128 x 128 and zoom 2.19. The planar gamma camera for scanning was with low energy, parallel hole, and general purpose collimator. Each frame acquisition counted 500 K. The energy window was maintained at 20% with the photopeak centered at 140 keV. Immediately after the delayed planar imaging (12), SPECT/CT integrated imaging was carried out for locating and differentiating diseased parathyroids. The choice of delayed phase for SPECT/CT imaging can reduce the rate of the high thyroid MIBI uptake during the early phase. Imaging data were reconstructed with a three-dimensional iterative algorithm. Images were smoothed using a Sharp(C) filter. Both SPECT and CT 3-mm slices were generated using an Astonish bone application package (Philips). SPECT/CT images were fused with Syntegra software V2.3.1 from Philips.

### The semiquantitative analysis of ^99m^Tc-MIBI image

**Figure 1:**
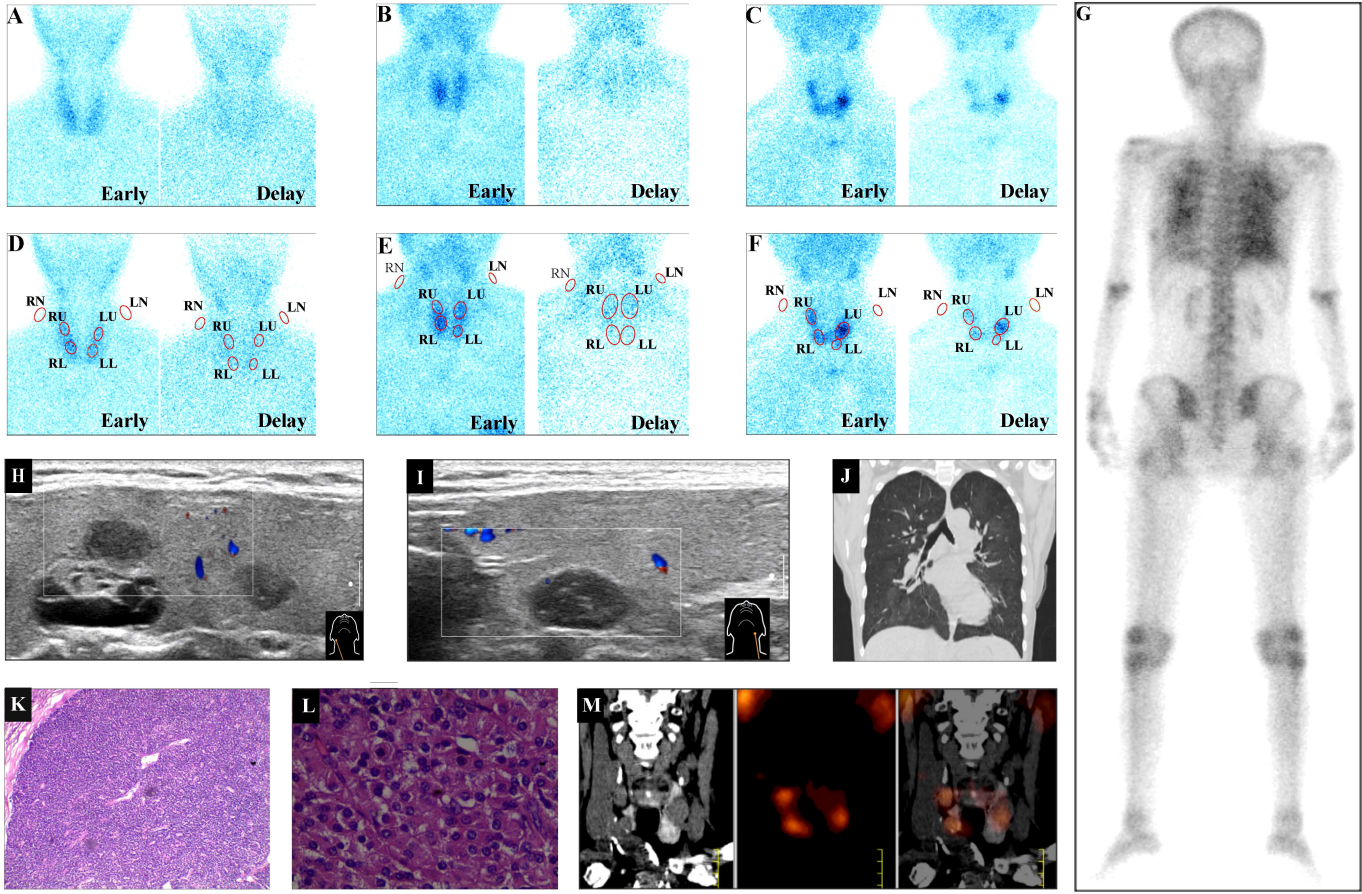
^99m^Tc-MIBI uptake intensity category examples. Based on the most intensive degree of ^99m^Tc-MIBI accumulation in parathyroids during the delayed phase, three levels of MIBI uptake comprising slight, medium, and high uptakes were illustrated. **(A)** The little MIBI concentration in all four parathyroid lobes illustrated the slight MIBI uptake. **(B)** The faint MIBI concentration in bilateral upper lobes and the right lower lobe illustrated the medium MIBI uptake. **(C)** The distinct MIBI concentration in bilateral upper lobes and the right lower lobe illustrated the high MIBI uptake. **(A)** and **(D)** (MIBI uptake) came from the same patient **(A), (B)** and **(E)** (MIBI uptake) and **(G**–**J)** came from the same patient **(B), (C)** and **(F)** (MIBI uptake) and **(K**–**M)** came from the same patient **(C)**. Red ellipses in **(D–F)** illustrated the ROIs of MIBI uptake in bilateral neck and four parathyroid lobes in **(A–C)**, respectively. **(G)** the posterior ^99m^Tc-MDP bone scan. **(H, I)** Hypoechoic ultrasound parathyroid images behind the right or left thyroid lobe, respectively. **(J)** CT scan image of thorax. **(K)** (H&E staining, × 40 original magnification) and **(L)** (H&E staining, × 200 original magnification), pathological images of left upper parathyroid adenoma from parathyroidectomy. **(M**) The coronal CT, coronal MIBI SPECT, and fused MIBI SPECT/CT image of bilateral parathyroids. MIBI, methoxyisobutylisonitrile; MDP, methyldiphosphate; RN, LN, right, left neck background, respectively; RU, LU, RL, LL, right upper, left upper, right lower, left lower parathyroid lobe, respectively; early, delay, early, delayed phase, respectively; H&E, hematoxylin and eosin.

For semiquantitatively analyzing the MIBI uptake, the regions of interest (ROIs) of parathyroids and bilateral neck were determined in software ImageJ 1.46r (National Institute of Health, USA). In the parathyroid images with medium or high MIBI uptake (defined in **Figure 1**), parathyroidal ROIs covered most areas with MIBI concentration. In the parathyroid MIBI scintigraphy with slight MIBI uptake (defined in **Figure 1**) and in the control group, parathyroidal ROIs covered most of the upper or lower one-third of the thyroid background image, which was referenced to the document (3), because the majority of parathyroids distributed among the four usual anatomical positions (6, 13). Most of the parathyroidal ROIs with slight MIBI uptake and all parathyroidal ROIs in the control group were referenced to the background of early-phase thyroid imaging. The parathyroidal ROIs with slight uptake in three cases were referenced to MIBI SPECT/CT imaging.

Based on ultrasound, CT, MRI, and/or SPECT/CT imaging, in the thyroid contour of the slight MIBI uptake group, the maximum nodule length was less than 9 mm, and there were 15 nodules with maximum nodule length ≥ 5 mm. In the 15 nodules, there were three that were in the middle of the thyroid contour described in ultrasound imaging, but there were no significant MIBI concentration in the areas of these three nodules; the other 12 nodules were all in the upper or lower one-third of thyroids contours. The following were parts of the reasons that all patients in the slight MIBI uptake group did not have pathological outcomes: ① the limited nodule length that determined the difficulty in biopsy; ② the ethical rules that routine biopsy of these patients was prohibited; ③ the comparably mild symptoms of SHPT, which determined that these patients were not willing to accept PTx.

In the ^99m^Tc-MIBI images of parathyroids, the blue dots represented the distribution of ^99m^Tc-MIBI (**Figure 1**). In ImageJ, the gray value mean in the area with few dots was greater than that in the area with many dots. The average value of the gray value mean of bilateral neck ROIs was utilized as the background. The calculation of the target (the gray value mean of a specified parathyroid ROI) to background ratio (TBR) was used as the indicator for semiquantitatively (14) manifesting the MIBI uptake strength. It can be assumed that in a ^99m^Tc-MIBI image of parathyroid, there was area A with fewer dots than area B, then the gray value mean and TBR in area A were greater than those in area B, i.e., the parathyroid TBR was negatively related to the MIBI uptake level. Therefore, the bigger the TBR, the less the MIBI uptake level; if some indices were positively related to TBR, these indices were negatively related to MIBI uptake intensity, and vice versa. MIBI washout rate = [(delayed TBR - early TBR) ×100]/delayed TBR.

For simplifying the expression of MIBI uptake and washout in parathyroid ROIs, the following variables were introduced: AvgE and AvgD, which were defined as the average value of four TBRs in parathyroid lobe ROIs of each patient during early and delayed phase, respectively; MinMeanE and MinMeanD, which were defined as the minimum one among the four TBRs in parathyroid lobe ROIs of each patient during early and delayed phase, respectively; MinWash and MaxWash, which were defined as the minimum and maximum one among the four parathyroid lobe washout rates, respectively.

### The visual category of ^99m^Tc-MIBI uptake intensity in individual parathyroid

Visually analyzing the MIBI uptake was reevaluated by two experienced nuclear medicine physicians blinded to laboratory, radiological, surgical, and pathological results. ^99m^Tc-MIBI uptake intensity in parathyroids was visually qualitatively graded into three levels (5), namely slight, medium, and high levels. Each level was based on the self-definition order of difficulty in discriminating diseased parathyroids. For expression convenience, the slight-, medium-, and high-level groups were named correspondingly as groups 1, 2, and 3, which were with little, faint, and distinct MIBI concentration in parathyroids, respectively. The three-level MIBI uptake is defined and illustrated in **Figure 1**, in which the parathyroidal MIBI uptake of patients A, B, and C was utilized as the example of the slight, medium, and high MIBI uptake levels, respectively.

Although patient A had a high serum PTH level of 739 pg/mL and CRF course 84 months, there was little or slight MIBI accumulation foci in the thyroid contour during early and delayed phases (**Figure 1A**); so the ROIs of four parathyroid lobes were determined based on a normal parathyroid anatomy (15) (**Figure 1D**).

Patient B had high serum PTH (377 pg/mL), CRF course 102 months, and enlarged parathyroid lobes behind the envelope of the right thyroid (**Figure 1H**) and the left thyroid (**Figure 1I**). In bilateral lungs, there were high ^99m^Tc-MDP uptake (**Figure 1G**) and high-density images (**Figure 1J**) suggesting ectopic calcinosis. Therefore, the moderate parathyroid MIBI uptake in bilateral upper lobes and the right lower lobe during the delayed phase (**Figure 1B**) should be enough to diagnose SHPT and localize the diseased parathyroids (**Figure 1E**).

Patient C had high serum PTH (2606 pg/mL) and CRF course 108 months. Therefore, the significant parathyroid MIBI uptake in bilateral upper lobes and the right lower lobe in the planar image (**Figure 1C**) and SPECT/CT images (**Figure 1M**) were enough to diagnose SHPT and localize diseased parathyroids (**Figure 1F**). The pathological results from PTx confirmed the high MIBI uptake foci to be parathyroid adenoma (**Figure 1K, L**).

### Statistical analysis

Data are shown as mean ± standard deviation (SD) for continuous variables. *P* < 0.05 was considered significant in this study. Chi-square analysis, one-way analysis of variance (ANOVA), independent samples *t*-test (both with two-tailed *P* value), correlation analysis with Pearson’s correlation coefficient and two-tailed *P* value, and multiple linear regression analysis were all performed with the statistical software Statistical Package for Social Sciences (SPSS, version 24, IBM, Armonk, USA). Receiver-operating characteristic (ROC) analysis was performed with MedCalc software (version 19.0.4, MedCalc Software bvba, Ostend, Belgium). In one-way ANOVA, *post hoc* test of Student–Newman–Keuls (SNK) or Games–Howell was used for homogeneous variances or heterogeneous variances, respectively.

## Results

### The average value of gray value mean in the ROIs of background areas

The average value of the gray value mean in the ROIs of right necks or left necks was 245.14 ± 6.18 or 244.45 ± 5.61 (cases = 151), respectively, during the early phase. The above average value of the gray value mean was 242.74 ± 8.05 or 241.34 ± 8.20 (cases = 151), respectively, during the delayed phase.

### The relativity of some indices to ^99m^Tc-MIBI uptake TBRs in CRF patients

The correlation analysis (**Tables 1** and **2**) of some indices related to CRF with such six MIBI uptake indicators, namely AvgE, MinMeanE, AvgD, MinMeanD, MinWash, and MaxWash, indicated the following: ① with the increment of patient age, CRF course, hemodialysis vintage, serum PTH, and AKP, MIBI uptake was increased; ② after oral administration of calcitriol and calcium (12) and with the increment of serum UA, MIBI uptake was decreased; ③ with the increment of patient age, MIBI washout was increased; ④ with the increment of serum phosphorus, Ca × P, and Vit B12, MIBI washout was decreased.

**Table 2:** The comparison of ^99m^Tc-MIBI uptake TBRs between different sex, thyroid function, or oral calcitriol.

Items		Sex		Thyroid function		Oral calcitriol	
		Women	Men	Low	Normal	No	Yes
Cases		75	76	38	113	41	110
AvgE	Mean	0.625	0.674	0.659	0.646	0.630	0.657
	*t*	-2.036		0.449		-1.012	
	*P*	**0.044**		0.654		0.313	
MinMeanE	Mean	0.542	0.591	0.579	0.562	0.520	0.584
	*t*	-1.647		0.480		-1.975	
	*P*	0.102		0.632		**0.049**	
AvgD	Mean	0.692	0.723	0.700	0.710	0.732	0.698
	*t*	-1.240		-0.361		1.169	
	*P*	0.217		0.718		0.244	
MinMeanD	Mean	0.623	0.674	0.635	0.654	0.645	0.650
	*t*	-1.648		-0.518		-0.150	
	*P*	0.102		0.605		0.881	
MinWash	Mean	-4.477	-8.593	-10.204	-5.319	1.272	-9.463
	*t*	0.782		-0.806		1.831	
	*P*	0.435		0.422		0.069	
MaxWash	Mean	16.720	15.720	12.232	17.556	22.832	13.750
	*t*	0.210		-0.973		1.712	
	*P*	0.834		0.332		0.089	

Oral calcitriol, oral administration of calcitriol and calcium; AvgE and AvgD, the average value of four TBRs in parathyroid ROIs of each patient during early and delayed phase, respectively; MinMeanE and MinMeanD, the minimum one among four TBRs in parathyroid ROIs of each patient during early and delayed phase, respectively; MinWash and MaxWash, the minimum and maximum one among four parathyroid washout ratios, respectively; t, independent samples Student’s t test; P, two-tailed P value. **Bold values** show significance after statistical analysis.

After multiple linear regression analysis, MIBI uptake may be predicted as

AvgE = 0.827 - 0.003 × age (years old) - 0.0001 × AKP (U/L), *R^2^* = 0.117, *F* = 9.371, *P* = 0.000, cases = 145;

MinMeanE = 1.019 - 0.005 × age (years old) - 0.0002 × AKP (U/L) + 0.087 × Ca (mmol/L), *R^2^* = 0.207, *F* = 12.251, *P* = 0.000, cases = 145;

AvgD = 0.816 - 0.002 × age (years old) + 0.0002 × UA (μmol/L) - 0.070 × phosphorus (nmol/L), *R^2^* = 0.100, *F* = 5.378, *P* = 0.002, cases = 150;

MinMeanD = 0.877 - 0.004 × age (years old) - 0.0002 × AKP (U/L) + 0.0003 × UA (μmol/L) - 0.081 × phosphorus (nmol/L), *R^2^* = 0.171, *F* = 7.234, *P* = 0.000, cases = 145;

### MIBI uptake discrepancy in different CRF course

The general tendency was that with CRF course increasing, MIBI uptake intensity was gradually increased (**Table 1**). However, the discrepancy of MIBI uptake intensity at a specific time point often emerged (**Supplementary Figure 1**).

### The comparison of ^99m^Tc-MIBI uptake intensity and some indices among three qualitative groups

If MIBI uptake was grouped on a per-parathyroid lobe basis, the MIBI uptake was significantly different among groups 1, 2, and 3 of all four parathyroids (*P* < 0.05). There was significantly more intensive MIBI uptake in group 3 than that in group 1 or 2 (*P* < 0.05) of most parathyroid lobes during the early or delayed phase. The MIBI uptake between groups 1 and 2 of most parathyroids was comparable (*P* > 0.05)(**Supplementary Table 1**).

If the MIBI uptake was grouped on a per-patient basis, all cases were divided into three groups based on the most intensive MIBI uptake level in four parathyroid lobes of every patient (**Figure 1**). The CRF course, hemodialysis vintage, AKP, Ca, CPI, and PTH were significantly greater (*P* < 0.05) in group 3 than in group 1 or 2 but were comparable (*P* > 0.05) between groups 1 and 2 during both the early (**Table 3**) and delayed phases (**Table 4**), except that CPI was significantly less (*P* < 0.05) in group 1 than in group 2 during the delayed phase.

**Table 3:** The comparison of some indices among three qualitative groups during early phase.

Indices	F	*P-1*	Group - 1		Group - 2		Group - 3		*Post hoc P-2*		
			Cases	Mean	Cases	Mean	Cases	Mean	① - ②	① - ③	② - ③
Age (years)	0.843	0.432	31	47.45	96	47.16	24	51.54	> 0.05	> 0.05	> 0.05
Course (months)	8.630	**0.000**	31	27.90	96	46.30	24	82.88	> 0.05	**< 0.05**	**< 0.05**
Hemodialysis months	20.563	**0.000**	31	15.42	96	18.66	24	74.29	> 0.05	**< 0.05**	**< 0.05**
GFR (ml/min)	1.680	0.201	10	25.27	24	16.85	3	12.67	> 0.05	> 0.05	> 0.05
AKP (U/L)	4.369	**0.014**	30	129.30	93	176.56	22	291.59	> 0.05	**< 0.05**	**< 0.05**
Creatinine (μmol/L)	1.060	0.349	31	930.02	96	847.81	23	946.62	> 0.05	> 0.05	> 0.05
BUN (mmol/L)	2.259	0.108	31	30.79	96	27.98	23	24.16	> 0.05	**< 0.05**	> 0.05
UA (μmol/L)	1.498	0.227	31	535.19	96	492.63	23	474.57	> 0.05	> 0.05	> 0.05
BUN/Creatinine	2.480	0.087	31	35.49	96	34.92	23	27.97	> 0.05	> 0.05	> 0.05
Ca (mmol/L)	7.258	**0.001**	31	2.00	96	2.13	24	2.40	> 0.05	**< 0.05**	**< 0.05**
Phosphorus (nmol/L)	0.318	0.728	31	1.90	96	1.99	24	1.95	> 0.05	> 0.05	> 0.05
Ca × P	2.772	0.066	31	3.79	96	4.21	24	4.64	> 0.05	**< 0.05**	> 0.05
CPI (mg/L)	5.227	**0.006**	31	6.24	92	6.65	22	7.90	> 0.05	**< 0.05**	**< 0.05**
Calcitonine (pg/mL)	1.701	0.186	31	3.59	96	6.37	24	9.51	> 0.05	> 0.05	> 0.05
PTH (pg/mL)	9.836	**0.000**	31	1056.4	96	1348.3	24	1919.2	> 0.05	**< 0.05**	**< 0.05**
Ferritin (ng/mL)	2.211	0.113	31	378.57	96	352.77	24	605.18	> 0.05	> 0.05	> 0.05
VitB12 (pmol/L)	0.814	0.445	31	443.55	96	439.79	24	523.42	> 0.05	> 0.05	> 0.05
Folate (nmol/L)	2.752	0.067	31	18.06	96	23.46	24	27.49	> 0.05	**< 0.05**	> 0.05
EPO (mIU/L)	1.427	0.243	30	20.32	90	36.75	24	62.43	> 0.05	> 0.05	> 0.05
Hb (g/L)	0.788	0.457	31	91.77	96	92.30	24	98.71	> 0.05	> 0.05	> 0.05

GFR, glomerular filtration rate; AKP, alkaline phosphatase; BUN, blood urea nitrogen; Ca, serum calcium ion; UA, uric acid; CPI, cysteine proteinase inhibitor C; PTH, parathyroid hormone; VitB12, vitamin B12; EPO, erythropoietin; Hb, hemoglobin. ①, group 1; ②, group 2; ③, group 3. F, P - 1, the F and P value for one-way ANOVA; P - 2, the P value for post hoc test. **Bold values** show significance after statistical analysis.

**Table 4:** The comparison of some indices among three qualitative groups during delayed phase.

Indices	F	*P - 1*	Group - 1		Group - 2		Group - 3		*Post hoc P - 2*		
			Cases	Mean	Cases	Mean	Cases	Mean	① - ②	① - ③	② - ③
Age (years)	0.691	0.503	41	47.98	90	47.10	20	51.45	> 0.05	> 0.05	> 0.05
Course (months)	5.415	**0.005**	41	49.47	90	46.32	20	81.15	> 0.05	**< 0.05**	**< 0.05**
Hemodialysis months	14.955	**0.000**	41	15.39	90	21.83	20	72.80	> 0.05	**< 0.05**	**< 0.05**
GFR (ml/min)	0.416	0.663	19	20.33	15	18.06	3	12.67	> 0.05	> 0.05	> 0.05
AKP (U/L)	4.861	**0.009**	40	141.50	87	176.79	18	315.17	> 0.05	**< 0.05**	**< 0.05**
Creatinine (μmol/L)	2.206	0.114	41	781.01	90	910.96	19	946.59	> 0.05	> 0.05	> 0.05
BUN (mmol/L)	1.746	0.178	41	27.70	90	29.01	19	23.66	> 0.05	> 0.05	> 0.05
UA (μmol/L)	2.051	0.132	41	535.17	90	487.67	19	471.89	> 0.05	> 0.05	> 0.05
BUN/Creatinine	4.254	**0.016**	41	38.50	90	33.25	19	27.62	> 0.05	**< 0.05**	**< 0.05**
Ca (mmol/L)	8.420	**0.000**	41	2.05	90	2.12	20	2.47	> 0.05	**< 0.05**	**< 0.05**
Phosphorus (nmol/L)	2.077	0.129	41	1.82	90	2.03	20	1.94	> 0.05	> 0.05	> 0.05
Ca × P	4.282	**0.016**	41	3.74	90	4.27	20	4.76	> 0.05	**< 0.05**	> 0.05
CPI (mg/L)	9.241	**0.000**	41	5.86	86	6.90	18	8.04	**< 0.05**	**< 0.05**	**< 0.05**
Calcitonine (pg/mL)	1.627	0.200	41	7.00	90	5.11	20	10.22	> 0.05	> 0.05	> 0.05
PTH (pg/mL)	5.188	**0.007**	41	1155.0	90	1385.6	20	1809.2	> 0.05	**< 0.05**	**< 0.05**
Ferritin (ng/mL)	1.685	0.189	41	337.52	90	382.04	20	595.18	> 0.05	> 0.05	> 0.05
VitB12 (pmol/L)	0.153	0.858	41	458.39	90	445.03	20	484.25	> 0.05	> 0.05	> 0.05
Folate (nmol/L)	1.082	0.342	41	20.78	90	23.14	20	26.89	> 0.05	> 0.05	> 0.05
EPO (mIU/L)	1.094	0.338	39	26.52	85	36.59	20	63.55	> 0.05	> 0.05	> 0.05
Hb (g/L)	1.471	0.233	41	93.12	90	91.44	20	101.35	> 0.05	> 0.05	> 0.05

GFR, glomerular filtration rate; AKP, alkaline phosphatase; BUN, blood urea nitrogen; Ca, serum calcium ion; UA, uric acid; CPI, cysteine proteinase inhibitor C; PTH, parathyroid hormone; VitB12, vitamin B12; EPO, erythropoietin; Hb, hemoglobin. ①, group 1; ②, group 2; ③, group 3. F, P - 1, the F and P value for one-way ANOVA; P - 2, the P value for post hoc test. **Bold values** show significance after statistical analysis.

Based on the above similarity and difference, groups 1 and 2 might be combined into one group. The combined new one group and the previous group 3 can be named as insignificant and significant groups, respectively, which were short for insignificant and significant MIBI uptake groups, respectively. The insignificant and significant groups constituted the two-level MIBI uptake groups.

### The comparison of ^99m^Tc-MIBI uptake TBRs and some indices between two qualitative groups based on two-level grouping method

The ROC analysis displayed the significant MIBI uptake difference (*P* < 0.05) between the insignificant and significant groups. The criteria (**Supplementary Table 2**) or optimal cutoff value (**Supplementary Figure 2**) of MIBI uptake TBR for differentiating the above two groups was 0.49–0.71 in an individual parathyroid lobe.

During early (**Table 5**) or delayed phase (**Table 6**), CRF course, hemodialysis vintage, serum AKP, Ca, CPI, and PTH were all significantly greater in the significant group than in the insignificant group (*P* < 0.05). BUN/creatinine was significantly less in the significant group than in the insignificant group (*P* < 0.05).

**Table 5:** The comparison of some indices between insignificant and significant MIBI uptake groups during early phase.

Indices	Insignificant group		Significant group		*t*	*P*
	Cases	Mean	Cases	Mean		
Age (years)	127	47.23	24	51.54	-1.300	0.196
Course (months)	127	41.81	24	82.88	-3.713	**0.000**
Hemodialysis months	127	17.87	24	74.29	-5.493	**0.000**
GFR (ml/min)	34	20.88	3	15.48	0.582	0.564
AKP (U/L)	123	165.03	22	291.51	-2.731	**0.007**
BUN (mmol/L)	127	28.67	23	24.16	1.750	0.082
Creatinine (μmol/L)	127	867.88	23	946.62	-0.957	0.340
UA (μmol/L)	127	503.02	23	474.57	0.897	0.371
BUN/Creatinine	127	35.06	23	27.97	2.226	**0.028**
Ca (mmol/L)	127	2.10	24	2.40	-3.481	**0.001**
Phosphorus (nmol/L)	127	1.96	24	1.95	0.143	0.886
Ca × P	127	4.11	24	4.64	-1.781	0.077
CPI (mg/L)	123	6.54	22	7.90	-3.064	**0.003**
Calcitonine (pg/mL)	127	5.69	24	9.51	-1.297	0.204
PTH (pg/mL)	127	1277.05	24	1919.15	-3.106	**0.004**
Ferritin (ng/mL)	127	359.07	24	605.18	-1.310	0.202
VitB12 (pmol/L)	127	440.71	24	523.42	-1.278	0.203
Folate (nmol/L)	127	22.14	24	27.49	-1.372	0.180
EPO (mIU/L)	120	32.64	24	62.43	-1.460	0.147
Hb (g/L)	127	92.17	24	98.71	-1.255	0.211

GFR, glomerular filtration rate; AKP, alkaline phosphatase; BUN, blood urea nitrogen; Ca, serum calcium ion; UA, uric acid; CPI, cysteine proteinase inhibitor C; PTH, parathyroid hormone; VitB12, vitamin B12; EPO, erythropoietin; Hb, hemoglobin; t, independent samples Student’s t test; P, two-tailed P value. **Bold values** show significance after statistical analysis.

**Table 6:** The comparison of some indices between insignificant and significant MIBI uptake groups during delayed phase.

Indices	Insignificant group		Significant group		*t*	*P*
	Cases	Mean	Cases	Mean		
Age (years)	131	47.37	20	51.45	-1.137	0.257
Course (months)	131	43.33	20	81.15	-3.132	**0.002**
Hemodialysis months	131	19.82	20	72.8	-5.410	**0.000**
GFR (ml/min)	34	19.33	3	12.67	0.793	0.433
AKP (U/L)	127	165.68	18	315.17	-2.979	**0.003**
BUN (mmol/L)	131	28.60	19	23.66	1.769	0.079
Creatinine (μmol/L)	131	870.29	19	946.59	-0.855	0.394
UA (μmol/L)	131	502.53	19	471.89	0.892	0.374
BUN/Creatinine	131	34.89	19	27.62	2.104	**0.037**
Ca (mmol/L)	131	2.10	20	2.47	-4.017	**0.000**
Phosphorus (nmol/L)	131	1.97	20	1.94	0.220	0.826
Ca × P	131	4.11	20	4.76	-2.017	**0.045**
CPI (mg/L)	127	6.57	18	8.04	-3.052	**0.003**
Calcitonine (pg/mL)	131	5.70	20	10.22	-1.327	0.198
PTH (pg/mL)	131	1313.44	20	1809.20	-2.755	**0.007**
Ferritin (ng/mL)	131	368.11	20	595.18	-1.091	0.288
VitB12 (pmol/L)	131	449.21	20	484.25	-0.500	0.618
Folate (nmol/L)	131	22.40	20	26.89	-1.037	0.311
EPO (mIU/L)	124	33.42	20	63.55	-1.369	0.173
Hb (g/L)	131	91.97	20	101.35	-1.677	0.096

GFR, glomerular filtration rate; AKP, alkaline phosphatase; BUN, blood urea nitrogen; Ca, serum calcium ion; UA, uric acid; CPI, cysteine proteinase inhibitor C; PTH, parathyroid hormone; VitB12, vitamin B12; EPO, erythropoietin; Hb, hemoglobin; t, independent samples Student’s t test; P, two-tailed P value. **Bold values** show significance after statistical analysis.

### The comparisons of some indices in control group with that in CRF patients

Between the control group and group 1, 2, or 3, the age was comparable (*F* = 0.461, *P* = 0.71, and after SNK *post hoc* test, all *P* > 0.940), and the gender was also comparable (Pearson’s χ^2^ = 3.87, *P* = 0.276). The serum AKP, BUN, Cre, UA, phosphorus, Ca × P, and PTH in the control group were all significantly less than that in group 1, 2, 3 or the insignificant group (all *P* = 0.000 in the ANOVA test). Both the serum BUN/Cre and Hb in the control group were significantly greater than those in group 1, 2, 3 or the insignificant group (all *P* = 0.000 in the ANOVA test). Serum calcium ions were 2.05 ± 0.28 mmol/L (cases = 41) in group 1, 2.12 ± 0.44 mmol/L (cases = 90) in group 2, 2.10 ± 0.40 mmol/L (cases = 131) in the insignificant group, 2.47 ± 0.32 mmol/L (cases =20) in group 3, and 2.27 ± 0.14 mmol/L (cases = 40) in the control group. Serum calcium ions in groups 1 and 2 and the insignificant group were all significantly less than those in the control group, all with *t* > 3.070 and *P* < 0.003. Serum calcium ions in group 3 were significantly greater than those in the control group (*t* = -2.600, *P* = 0.016).

### The relativity of some indices to ^99m^Tc-MIBI uptake intensity in CRF patients plus control group

In the cohort of CRF patients and the control group (**Supplementary Table 3**), subjects’ age, serum AKP, BUN, creatinine, phosphorus, Ca × P, and PTH were significantly positively related to the MIBI uptake (*P* < 0.05), but serum BUN/Cre was significantly negatively related to the MIBI uptake (*P* < 0.05). Serum BUN, creatinine, phosphorus, PTH, and Ca × P were significantly negatively related to MIBI washout (*P* < 0.05), but subjects’ age and serum BUN/Cre were significantly positively related to MIBI washout (*P* < 0.05).

### The comparisons of ^99m^Tc-MIBI uptake TBRs between control group and other groups

The MIBI uptakes in groups 1, 2, and 3 and the insignificant group during the delayed phase and in group 3 during the early phase were significantly greater than that in the control group (*P* < 0.05). MinWash in groups 1, 2, and 3 and the insignificant group were all significantly less than that in the control group (*P* < 0.05). MaxWash in group 2 and in the insignificant group were both significantly less than that in the control group (*P* < 0.05) (**Supplementary Table 4**).

### The correlations of both indices

All listed indices relative to CRF were positively or negatively significantly related to some other indices (*P* < 0.05) (**Supplementary Table 5**), partly similar to documented reports (5). Especially serum calcium was irrelative to the MIBI uptake in the parathyroids of CRF patients (**Table 1**, **Supplementary Table 3**), but serum calcium was significantly related to CRF course, hemodialysis vintage, serum BUN, CPI, and Hb.

## Discussion

The following pieces of evidence proved that all the methods in this paper were reliable: ① As the background for the calculation of MIBI uptake TBRs in parathyroids, the gray value of bilateral neck with little variance should be superior to that of thyroids (7) with big variance. ② The visual category of MIBI uptake intensity in every parathyroid lobe was in consensus between two experienced nuclear medicine physicians, which reduced the subjectivity in category. ③ The ROI areas of parathyroids in group 1 and the control group could not be determined accurately depending on planar MIBI imaging, but the determination methods of these areas were referenced to several previous documents (3, 6, 13). ④ It is possible that the ROI areas of parathyroids in groups 1–3 might be the ROI areas of other tissues including thyroid cancer or lymph nodes because there were limited pathological outcomes from PTx, but the following pieces of evidence rigorously prove that the ROI areas for parathyroids were mostly parathyroidal ROIs: (1) in the parathyroid ROI areas of groups 1, 2, and 3, the MIBI uptake was significantly increased, the MIBI washout was significantly decreased (**Supplementary Table 4**), and the MIBI uptake was significantly related to CRF indices such as serum AKP, BUN, creatinine, BUN/Cre, UA, phosphorus, and Ca × P (**Supplementary Table 3**); (2) the serum PTH in groups 1, 2, and 3 was significantly increased compared with that in the control group, and serum PTH was gradually increased from groups 1 to 3 (**Tables 3** and **4**). Although thyroid cancer or lymph nodes can also increase the MIBI uptake and delay the MIBI washout, the serum PTH and other indices related to CRF in patients with thyroid cancer or enlarged lymph nodes in thyroids cannot be significantly relatedly elevated.

CRF will lead to many abnormal biochemical indices. In CRF patients, the retention of phosphorus and other metabolite and the reduction of calcium and vitamin D levels stimulate the synthesis and secretion of PTH as well as the proliferation or nodularization rate of parathyroid cells (16). Therefore, it is essential to explore the relativity of some factors to the MIBI uptake in parathyroids to find the relevant factors to improve the diagnostic accuracy of the MIBI uptake. These relevant factors might be beneficial to predict the MIBI uptake intensity in parathyroids of SHPT patients.

Calcium is one of the important factors influencing serum PTH concentration (16). **Table 1** shows that serum calcium levels were irrelative to the MIBI uptake in the parathyroids of CRF patients, which may be relative to the oral administration of calcitriol and calcium (**Table 2**), similar to Torregrosa’s study (17). However, **Tables 3**, **4**, **5**, and **6** show that serum calcium was significantly different between groups 1 and 3, between groups 2 and 3, and between the insignificant and significant groups. The different results might be related to the fact that low serum calcium ions originated from early CRF because serum calcium concentrations were significantly positively relative to the CRF course, hemodialysis months, and serum Hb, BUN, and CPI concentration (**Supplementary Table 5**). Early SHPT with low serum calcium gradually developed into tertiary hyperparathyroidism, which will increase serum calcium ions. In clinical practice, it is impossible to stop reasonable treatments, including relieving the symptoms related to low serum calcium ion, because that will violate ethical rules; therefore, it is essential to explore other indicators related to the MIBI uptake in the parathyroids of CRF patients.

In this paper, 23 items closely relevant to CRF were studied. There were many factors that can significantly affect the MIBI uptake in parathyroids (3), but several factors were not significantly associated with the MIBI uptake, although the irrelevance remained elusive (18). Some indices can directly predict MIBI uptake intensity, while other indices cannot directly predict MIBI uptake intensity. These indicated the complexity, interaction, and even mutual offset of these indices in influencing the MIBI uptake. Therefore, it is essential to more deeply investigate the relation of some factors related to CRF to the MIBI uptake in parathyroids. Usually with the CRF progress, the stimulated parathyroid mainly by low serum calcium will secrete more and more PTH, following which the MIBI uptake in parathyroid lobes was strengthened. However, the MIBI uptake tendency in anyone of the four parathyroid lobes can be changed, even fluctuating, whatever during the early or delayed phase (**Supplementary Figure 1**), similar to Mario’s findings (19). The treatment for CRF included hemodialysis, PTx, supplement of EPO, calcitriol and calcium, levothyroxine, calcitonine, biphosphate, calcimimetic agent cinacalcet, and so on. These treatments can change serum calcium concentration and interfere with the proliferation of parathyroids and the MIBI uptake in parathyroids. EPO was mainly secreted by the kidneys, so CRF will decrease the EPO and the corresponding hematopoietic function. Although EPO was not directly related to the MIBI uptake, it can be significantly related to several indices relevant to CRF (**Supplementary Table 5**). Other factors such as serum ferritin, folate, Vit B12, and Hb were also relative to hematopoietic functions and chronic renal failure. These factors displayed similar phenomena to EPO in relation to the MIBI uptake in parathyroids.

It will be very important to reasonably utilize some serum indices related to CRF as parameters to construct a reliable formula to analyze the MIBI uptake in parathyroids as easily as possible because that will benefit surgeons to increase the success rate of surgical resection of diseased parathyroids. However, the presumption should be verified later.

The uptake mechanism of ^99m^Tc-MIBI by parathyroids has been proposed to occur based on the increased vascularity, capillary permeability and metabolism, mitochondrial entrapment in oxyphilic cells, the active phase of parathyroid cells (20, 21), p-glycoprotein or multidrug resistance-related protein expression (7, 18), parathyroid lesion weight, single or multiple parathyroid lesions, presence of parathyroid cyst (12), etc. MIBI washes out more rapidly from the thyroid than from the hyperfunction parathyroid, which is the basic principle for dual-phase parathyroidal imaging (22). Therefore, the diagnostic efficiency of diseased parathyroids with MIBI imaging changed with big variance (4, 9, 23).

Through comparing MIBI uptake intensity and multiple factors related to CRF by three- and two-level grouping methods (**Tables 3**, **4**, **5**, and **6**), the medium group should not be kept, even if it was possible that the rate of missing the diagnosis of the positive MIBI uptake was elevated, but the possibility was statistically little. Physicians were afraid of missing possible positive cases in group 2 but had no confidence in localizing the abnormal parathyroid lobe only depending on the MIBI uptake with an inexplicit outline. The MIBI uptake mean values were all less in group 1 than in group 2, although the MIBI uptake TBRs were statistically comparable (*P* > 0.05) between them (**Supplementary Table 1**). This indicated that the nuclear medicine physicians had chosen the appropriate cases into corresponding groups, although the determination was hard. By dichotomizing the patient MIBI uptake level outcomes into insignificant and significant groups, the two-level grouping method did not sacrifice analytical rigor; on the contrary, in clinical practice, the diagnostic efficiency and accuracy should be improved without loss of diagnostic confidence because between these two groups, there were significantly different MIBI uptake, biochemical data, and time indices related to CRF progression and treatment. Therefore, the two-level grouping method should be recommended to be adopted in clinical practice for visually discriminating the MIBI uptake intensity.

It was amazing that it did not seem that there was visual MIBI concentration in the MIBI scintigraphy of group 1, but TBRs in the parathyroid ROIs of group 1 were significantly less than those of the control group (**Supplementary Table 4**), which indicated that there was significantly more MIBI uptake and retention in the parathyroid ROIs of group 1 than those of the control group. The increased MIBI uptake was significantly related to the increased serum PTH and other indices related to CRF (**Table 3** and **4**), which indicated that the increased MIBI uptake in ROIs should be related to SHPT because of CRF. Therefore, semiquantitative assessment of the MIBI uptake was more sensitive than the visual assessment of the MIBI uptake in the parathyroids of CRF patients, which indicated that semiquantitative assessment of the MIBI uptake in group 1 might be promising for broader application.

To our knowledge, this paper firstly emphasized on the following: ① the roles of 23 items related to CRF in widening the view of exploring the correlation of the MIBI uptake with clinical practice (5); ② MIBI uptake tendency in parathyroids might alter; ③ the correlation of the most intensive MIBI uptake level with some indices related to CRF might be stronger than the correlation of the average MIBI uptake value (5) with these indices because more indices showed stronger relativity to MinMeanE than AvgE, and more indices showed stronger relativity to MinMeanD than AvgD (**Table 1**, **Supplementary Table 3**); ④ the two-level grouping method of the MIBI uptake can preserve the qualitatively diagnostic accuracy and improve diagnostic efficiency.

The main limitation of this paper lied on that only part of patients who had pathological outcomes from PTx or had undertaken MIBI SPECT/CT imaging, ultrasound, CT, and/or MRI for parathyroid location, although they were considered to be not essential (7, 13, 24) because of their limited accuracy in localizing tertiary parathyroids. The following measurements should mitigate the bias in parathyroid(s) localization: ① the strict patient enrollment criteria; ② the persuasive determination of ROIs in the background and parathyroids; ③ the introduction of the control group for comparing and correlating the MIBI uptake in ROIs to several indices related to CRF; ④ the large patient population; and ⑤ the significant relativity of the MIBI uptake in ROIs to serum PTH, CRF course, and other serum indices related to CRF.

## Conclusions

Parathyroids themselves and at least nine indices related to CRF can significantly influence the MIBI uptake in the parathyroids of SHPT patients, but the influential tendency sometimes varied. The two-level grouping method consisting of insignificant and significant MIBI uptake groups should be suitable for the qualitative diagnosis of the MIBI uptake.

## Data availability statement

The original contributions presented in the study are included in the article/**Supplementary Materials**. Further inquiries can be directed to the corresponding authors.

## Ethics statement

The studies involving human participants were reviewed and approved by the review committee of the First People’s Hospital of Yunnan Province. Written informed consent to participate in this study was provided by the participants’ legal guardian/next of kin.

## Author contributions

DY, LZ, YaJ, and YoJ conceptualized the paper. DY, YaJ, MXW, XQW, LJZ, MKW, and YoJ evaluated and reported the imaging findings. DY, LZ, and YaJ drafted and edited the manuscript. YoJ reviewed the manuscript. All authors acquired, analyzed, and interpreted data, commented on and revised the paper, and approved the final version of the manuscript.

## Acknowledgments

We thank the Laboratory Department and Nephrological Department in the First People’s Hospital of Yunnan Province for their help with biochemical tests and clinical service.

## Conflict of interest

The authors declare that the research was conducted in the absence of any commercial or financial relationships that could be construed as a potential conflict of interest.

## Publisher’s note

All claims expressed in this article are solely those of the authors and do not necessarily represent those of their affiliated organizations, or those of the publisher, the editors and the reviewers. Any product that may be evaluated in this article, or claim that may be made by its manufacturer, is not guaranteed or endorsed by the publisher.

## Abbreviations

AKP, alkaline phosphatase; ANOVA, one way analysis of variance; AvgD, the average value of four TBRs in parathyroid ROIs during the delayed phase; AvgE, the average value of four TBRs in parathyroid ROIs during the early phase; BUN, blood urea nitrogen; Ca, calcium; CPI, cysteine proteinase inhibitor C; Cre, creatinine; CRF, chronic renal failure; CT, computed tomography; EPO, erythropoietin; Fer, ferritin; Hb, hemoglobin; LL, left lower parathyroid; LN, left neck; LU, left upper parathyroid; MaxWash, the maximum one among four parathyroid washout ratios; MIBI, methoxyisobutylisonitrile; MinMeanD, the minimum one among four TBRs in parathyroid ROIs during the delayed phase; MinMeanE, the minimum one among four TBRs in parathyroid ROIs during the early phase; MinWash, the minimum one among four parathyroid washout ratios; MRI, magnetic resonance imaging; P, phosphorus; PTH, parathyroid hormone; PTx, parathyroidectomy; RL, right lower parathyroid; RN, right neck; ROC, receiver-operating characteristic; ROIs, regions of interest; RU, right upper parathyroid; SD, standard deviation; SHPT, secondary hyperparathyroidism; SPECT, single photon emission computed tomography; TBR, target to background ratio; UA, uric acid; VitB12, vitamin B12.
