# Supplementary figures and images for: Semiquantitative assessment of 99mTc-MIBI uptake in parathyroids of secondary hyperparathyroidism patients with chronic renal failure
Source: Front Endocrinol (Lausanne). 2022 Sep 8;13:915279. doi: 10.3389/fendo.2022.915279 (PMC9492857; doi:10.3389/fendo.2022.915279)

Supplementary Figure 1 MIBI uptake change

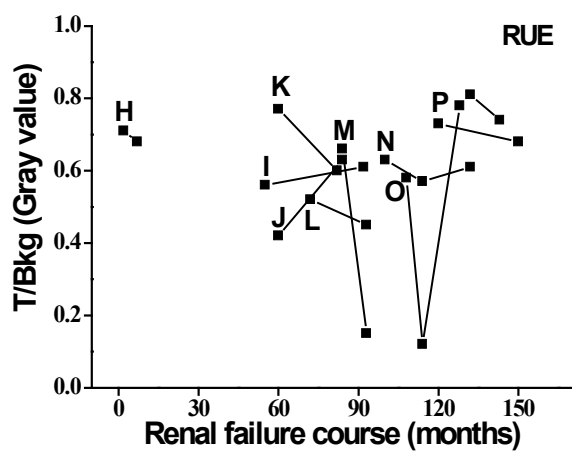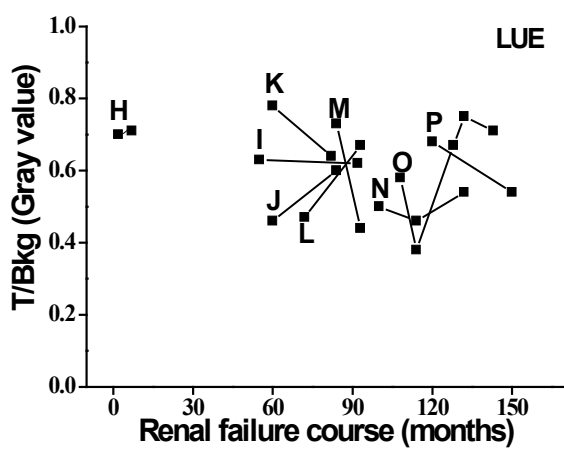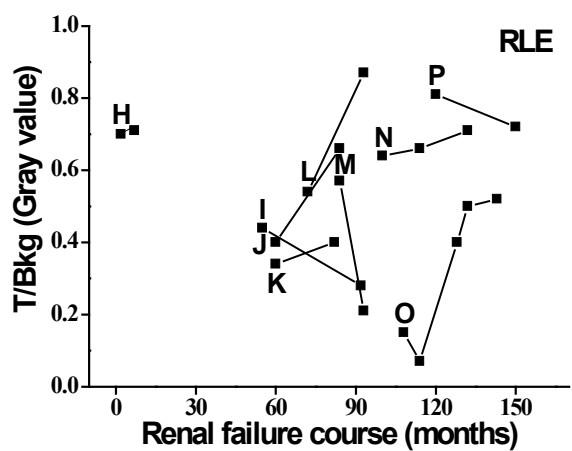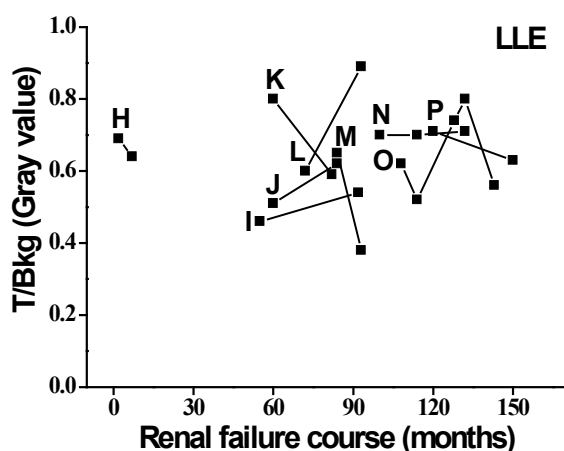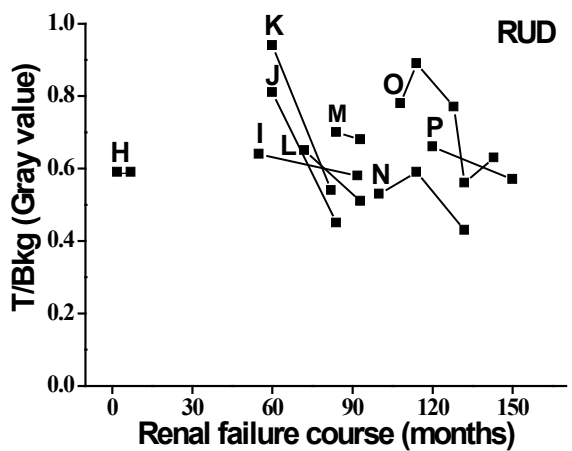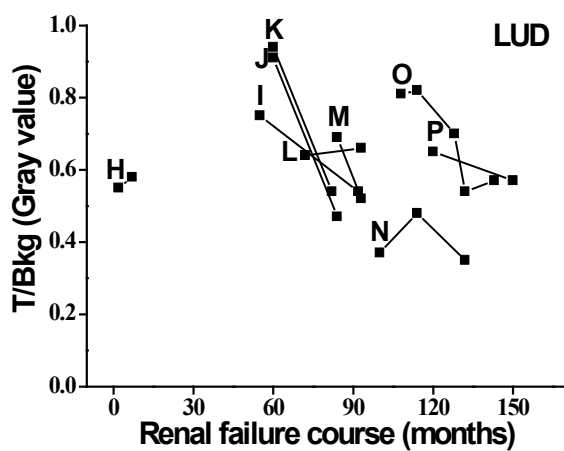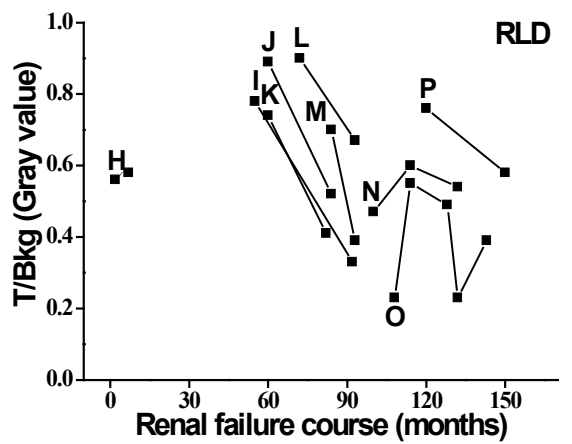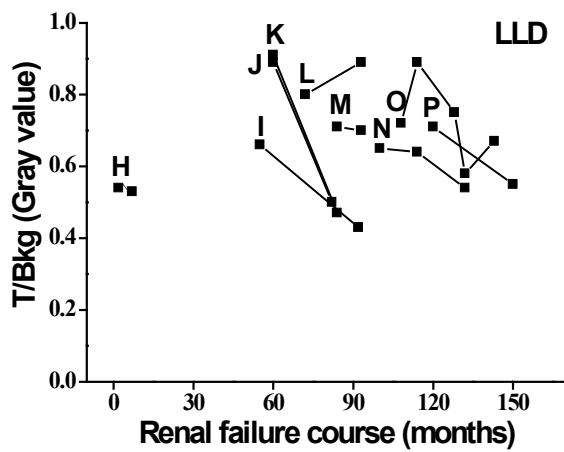

Supplement: Supplementary file 1 [file DataSheet_1.pdf]

# Supplementary Figure 2    ROC analysis of MIBI uptake

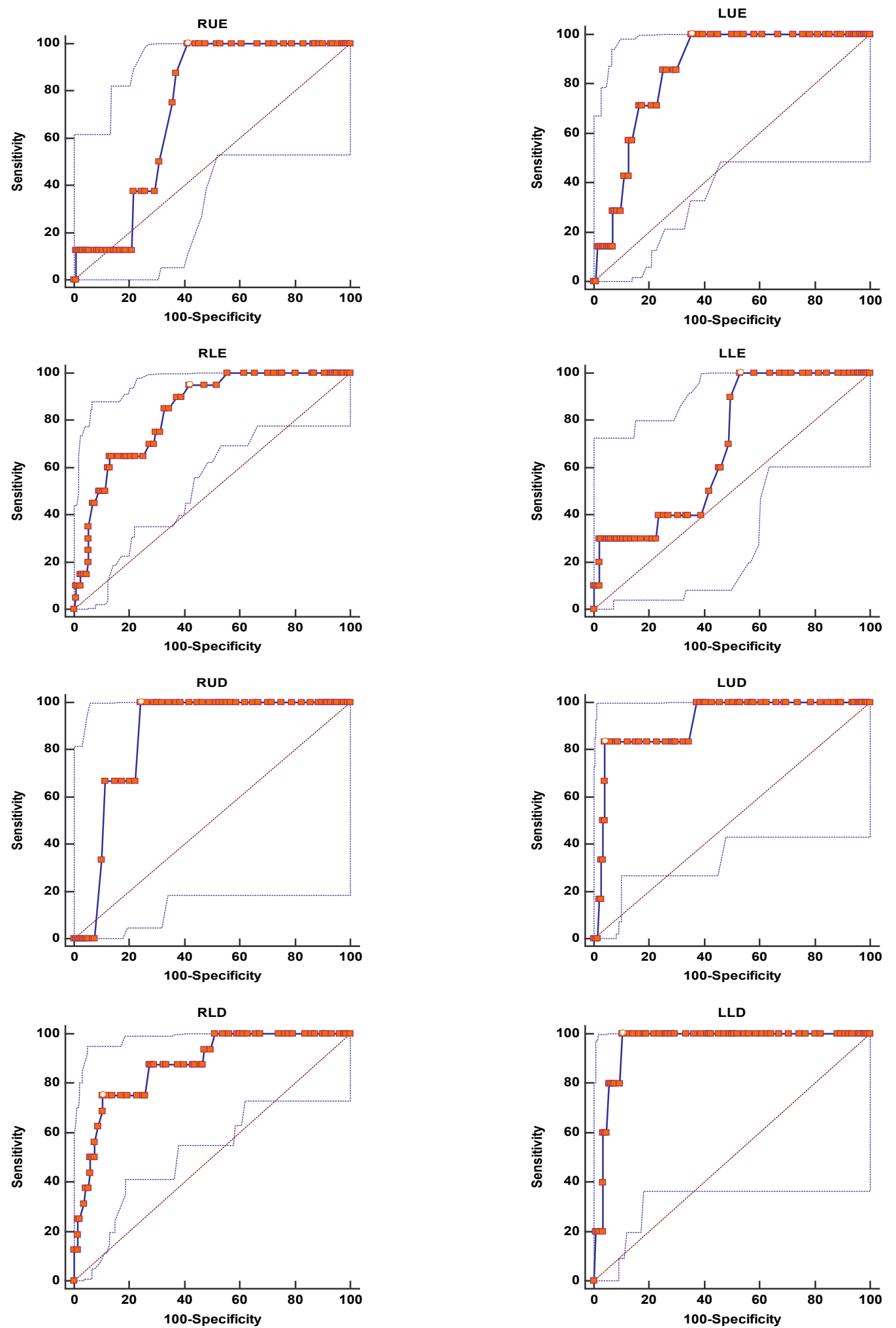

Supplement: Supplementary file 2 [file DataSheet_2.pdf]
