# Supplementary material for: Semiquantitative assessment of 99mTc-MIBI uptake in parathyroids of secondary hyperparathyroidism patients with chronic renal failure
Source: Front Endocrinol (Lausanne). 2022 Sep 8;13:915279. doi: 10.3389/fendo.2022.915279 (PMC9492857; doi:10.3389/fendo.2022.915279)
Supplement: Supplementary file 3 [file DataSheet_3.docx]

Legends-supplementary materials

**Supplementary Figure 1** ^99m^Tc-MIBI uptake change in different renal failure course

Letter H-P represent 9 patients, and the same letter represents the same patient. **T**, the gray value of MIBI uptake in each parathyroid lobe; **Bkg**, the average gray value of MIBI uptake in the ROIs of bilateral neck; **RUE**, **RLE**, **LUE**, **LLE**, right upper, right lower, left upper, left lower parathyroid lobe during early phase, respectively; **RUD**, **LUD**, **RLD**, **LLD**, right upper, left upper, right lower, left lower parathyroid lobe during delayed phase, respectively.

**Supplementary Table 1** the gray value comparison of ^99m^Tc-MIBI uptake among 3 qualitative groups

**RUE**, **RLE**, **LUE**, **LLE**, right upper, right lower, left upper, left lower parathyroid lobe during early phase, respectively; **RUD**, **LUD**, **RLD**, **LLD**, right upper, left upper, right lower, left lower parathyroid lobe during delayed phase, respectively. **Group 1**, **2** and **3**, slight, medium and high MIBI uptake group, respectively; **①**, group 1; **②**, group 2; **③**, group 3. **F, *P - 1***, the F and P value for one-way ANOVA; ***P - 2***, the P value for *post-hoc* test. **Bold values**, show significance after statistical analysis.

**Supplementary Table 2** ROC characters of ^99m^Tc-MIBI uptake TBRs for differentiating insignificant from significant MIBI uptake group

**RUE**, **RLE**, **LUE**, **LLE**, right upper, right lower, left upper, left lower parathyroid lobe during early phase, respectively; **RUD**, **LUD**, **RLD**, **LLD**, right upper, left upper, right lower, left lower parathyroid lobe during delayed phase, respectively; **criteria**, the optimal cutoff of MIBI uptake TBRs for differentiating insignificant from significant MIBI uptake group; **AUC**, the area under the curve; **ROC**, receiver operating characteristic curve. **Z**, z statistic; **J**, Youden index. **Bold values**, show significance after statistical analysis.

**Supplementary Figure 2** ROC analysis of ^99m^Tc-MIBI uptake in 4 parathyroids in CRF patients

The red circles in all figures indicate the optimal cutoff value of MIBI uptake TBRs for differentiating the insignificant from significant MIBI concentration in parathyroid lobes. **RUE**, **RLE**, **LUE**, **LLE**, right upper, right lower, left upper, left lower parathyroid lobe during early phase, respectively; **RUD**, **LUD**, **RLD**, **LLD**, right upper, left upper, right lower, left lower parathyroid lobe during delayed phase, respectively. The fluctuating dashed lines in ROC graphs indicate 95%CI of AUC. **ROC**, receiver operating characteristic curve; **CI**, confidence interval; **AUC**, area under curve.

**Supplementary Table 3** the relativity of some indices to ^99m^Tc-MIBI uptake TBRs in the cohort of control group and CRF patients

**AvgE**, **AvgD**, the average value of 4 TBRs in parathyroid lobes ROIs of each patient during early, delayed phase, respectively; **MinMeanE**, **MinMeanD**, the minimum one among 4 TBRs in parathyroid lobes ROIs of each patient during early, delayed phase, respectively; **MinWash**, **MaxWash**, the minimum, maximum one among 4 parathyroid lobes washout ratios, respectively; **AKP**, alkaline phosphatase; **BUN**, blood urea nitrogen; **Ca**, serum calcium ion; **UA**, uric acid; **PTH**, parathyroid hormone; P, phosphorus; **Hb**, hemoglobin. Pearson’s correlation was performed for analyses. *^a^P* < 0.05. *^b^P* < 0.01. *^c^P* < 0.001. **Bold values**, show significance after statistical analysis.

**Supplementary Table 4** the comparisons of ^99m^Tc-MIBI uptake TBRs between control group and other groups

The cases in control group, group 1, 2 and 3, and insignificant group were 40, 41, 90, 20 and 131, respectively. Insignificant group = group 1 + group 2; **AvgE**, **AvgD**, the average value of 4 TBRs in parathyroid lobes ROIs of each patient during early, delayed phase, respectively; **MinMeanE**, **MinMeanD**, the minimum one among 4 TBRs in parathyroid lobes ROIs of each patient during early, delayed phase, respectively; **MinWash**, **MaxWash**, the minimum, maximum one among 4 parathyroid lobes washout ratios, respectively. ***t***, independent samples *student*’s *t* test; ***P***, 2-tailed P value; **SD**, standard deviation. **Bold values**, show significance after statistical analysis.

**Supplementary Table 5** The correlations of both indices in CRF patients

**GFR**, glomerular filtration rate; **AKP**, alkaline phosphatase; **BUN**, blood urea nitrogen; **Cre**, creatinine; **Ca**, serum calcium ion; **P**, phosphorus; **UA**, uric acid; **CPI**, cysteine proteinase inhibitor C; **PTH**, parathyroid hormone; **VitB12**, vitamin B12; **EPO**, erythropoietin; **Hb**, hemoglobin. ***r***, Pearson’s relativity; ***P***, 2-tailed P value. *^a^P* < 0.05. *^b^P* < 0.01. *^c^P* < 0.001. **Bold values**, show significance after statistical analysis.
