# Supplementary material for: Semiquantitative assessment of 99mTc-MIBI uptake in parathyroids of secondary hyperparathyroidism patients with chronic renal failure
Source: Front Endocrinol (Lausanne). 2022 Sep 8;13:915279. doi: 10.3389/fendo.2022.915279 (PMC9492857; doi:10.3389/fendo.2022.915279)
Supplement: Supplementary file 4 [file Table_1.docx]

**Supplementary Table 1** the comparison of ^99m^Tc-MIBI uptake TBRs among 3 qualitative groups

| parathyroid lobe | F | *P - 1* | group - 1 | |  | group - 2 | |  | group - 3 | |  | *post-hoc P - 2* | | |
| --- | --- | --- | --- | --- | --- | --- | --- | --- | --- | --- | --- | --- | --- | --- |
|  |  |  | cases | mean |  | cases | mean |  | cases | mean |  | ① - ② | ① - ③ | ② - ③ |
| RUE | 6.139 | **0.003** | 74 | 0.693 |  | 69 | 0.625 |  | 8 | 0.546 |  | > 0.05 | **< 0.05** | > 0.05 |
| RLE | 20.624 | **0.000** | 72 | 0.704 |  | 59 | 0.614 |  | 20 | 0.443 |  | **0.005** | **0.000** | **0.005** |
| LUE | 5.818 | **0.004** | 59 | 0.675 |  | 85 | 0.642 |  | 7 | 0.458 |  | > 0.05 | **< 0.05** | **< 0.05** |
| LLE | 5.833 | **0.004** | 73 | 0.695 |  | 68 | 0.649 |  | 10 | 0.521 |  | 0.164 | 0.145 | 0.316 |
| RUD | 4.326 | **0.015** | 101 | 0.739 |  | 47 | 0.683 |  | 3 | 0.542 |  | 0.073 | **0.007** | **0.016** |
| RLD | 17.829 | **0.000** | 87 | 0.729 |  | 48 | 0.703 |  | 16 | 0.457 |  | > 0.05 | **< 0.05** | **< 0.05** |
| LUD | 8.803 | **0.000** | 85 | 0.735 |  | 60 | 0.707 |  | 6 | 0.461 |  | > 0.05 | **< 0.05** | **< 0.05** |
| LLD | 10.409 | **0.000** | 79 | 0.718 |  | 67 | 0.721 |  | 5 | 0.379 |  | > 0.05 | **< 0.05** | **< 0.05** |
