# Supplementary material for: Semiquantitative assessment of 99mTc-MIBI uptake in parathyroids of secondary hyperparathyroidism patients with chronic renal failure
Source: Front Endocrinol (Lausanne). 2022 Sep 8;13:915279. doi: 10.3389/fendo.2022.915279 (PMC9492857; doi:10.3389/fendo.2022.915279)
Supplement: Supplementary file 5 [file Table_2.docx]

**Supplementary Table 2** ROC characteristics of MIBI uptake TBRs for differentiating insignificant from significant MIBI uptake group

| parathyroid lobe | significant cases | insignificant cases | AUC | Z | *P* | criteria | J | sensitivity (%) | specificity (%) |
| --- | --- | --- | --- | --- | --- | --- | --- | --- | --- |
| RUE | 8 | 143 | 0.731 | 4.299 | **< 0.0001** | ≤ 0.64 | 0.5874 | 100.00 | 58.74 |
| RLE | 20 | 131 | 0.834 | 8.207 | **< 0.0001** | ≤ 0.68 | 0.5302 | 95.00 | 58.02 |
| LUE | 7 | 144 | 0.853 | 7.700 | **< 0.0001** | ≤ 0.62 | 0.6458 | 100.00 | 64.58 |
| LLE | 10 | 141 | 0.691 | 2.575 | **0.0100** | ≤ 0.71 | 0.4681 | 100.00 | 46.81 |
| RUD | 3 | 148 | 0.857 | 6.914 | **< 0.0001** | ≤ 0.59 | 0.7568 | 100.00 | 75.68 |
| RLD | 16 | 135 | 0.870 | 8.509 | **< 0.0001** | ≤ 0.50 | 0.6463 | 75.00 | 89.63 |
| LUD | 6 | 145 | 0.913 | 7.328 | **< 0.0001** | ≤ 0.49 | 0.7920 | 83.33 | 95.86 |
| LLD | 5 | 146 | 0.955 | 22.116 | **< 0.0001** | ≤ 0.51 | 0.8973 | 100.00 | 89.73 |
