# Supplementary material for: Semiquantitative assessment of 99mTc-MIBI uptake in parathyroids of secondary hyperparathyroidism patients with chronic renal failure
Source: Front Endocrinol (Lausanne). 2022 Sep 8;13:915279. doi: 10.3389/fendo.2022.915279 (PMC9492857; doi:10.3389/fendo.2022.915279)
Supplement: Supplementary file 6 [file Table_3.docx]

**Supplementary Table 3** the relativity of some indices to ^99m^Tc-MIBI uptake TBRs in the cohort of control group and CRF patients

| indices(unit) | normal range | mean | cases | AvgE | MinMeanE | AvgD | MinMeanD | MinWash | MaxWash |
| --- | --- | --- | --- | --- | --- | --- | --- | --- | --- |
| age (years) | 14 -78 | 47.87 | 191 | **-0.276^c^** | **-0.284^c^** | **-0.155^a^** | **-0.210^b^** | -0.027 | **0.160^a^** |
| AKP (U/L) | 35 -104 | 168.96 | 169 | -0.130 | **-0.229^b^** | -0.134 | **-0.216^b^** | -0.034 | 0.091 |
| BUN (mmol/L) | 2.0 - 7.1 | 23.38 | 188 | 0.024 | -0.019 | **-0.165^a^** | -0.134 | **-0.216^b^** | -0.104 |
| Creatinine (μmol/L) | 45 - 84 | 715.79 | 188 | 0.007 | -0.065 | **-0.229^b^** | **-0.213^b^** | **-0.227^b^** | -0.126 |
| UA (μmol/L) | 178 - 416 | 467.75 | 188 | 0.089 | 0.042 | 0.050 | 0.062 | -0.043 | -0.023 |
| BUN/Creatinine |  | 43.21 | 188 | 0.007 | 0.086 | **0.193^b^** | **0.215^b^** | **0.173^a^** | 0.091 |
| Ca (mmol/L) | 2.1 - 2.6 | 2.17 | 191 | -0.098 | -0.129 | 0.009 | -0.064 | 0.074 | 0.072 |
| phosphorus (nmol/L) | 0.8 - 1.45 | 1.81 | 180 | 0.051 | 0.001 | **-0.226^b^** | **-0.196^b^** | **-0.292^c^** | **-0.181^a^** |
| Ca × P |  | 0.65 | 180 | 0.006 | -0.063 | **-0.205^b^** | **-0.213^b^** | **-0.248^b^** | -0.137 |
| PTH (pg/mL) | 15 - 68.3 | 1107.86 | 191 | -0.039 | **-0.198^b^** | **-0.200^b^** | **-0.295^c^** | **-0.209^b^** | -0.020 |
| Hb (g/L) | 110 - 150 | 103.46 | 191 | -0.065 | 0.005 | 0.059 | 0.072 | 0.108 | 0.054 |
