# Supplementary material for: Semiquantitative assessment of 99mTc-MIBI uptake in parathyroids of secondary hyperparathyroidism patients with chronic renal failure
Source: Front Endocrinol (Lausanne). 2022 Sep 8;13:915279. doi: 10.3389/fendo.2022.915279 (PMC9492857; doi:10.3389/fendo.2022.915279)
Supplement: Supplementary file 7 [file Table_4.docx]

**Supplementary Table 4** the comparisons of ^99m^Tc-MIBI uptake TBRs between control group and other groups

| MIBI uptake | group | Mean | SD | *t* | *P* |  | MIBI uptake | group | Mean | SD | *t* | *P* |
| --- | --- | --- | --- | --- | --- | --- | --- | --- | --- | --- | --- | --- |
| AvgE | control | 0.652 | 0.095 |  |  |  | AvgD | control | 0.791 | 0.072 |  |  |
|  | 1 | 0.665 | 0.172 | -0.409 | 0.684 |  |  | 1 | 0.729 | 0.171 | 2.141 | **0.037** |
|  | 2 | 0.660 | 0.126 | -0.361 | 0.719 |  |  | 2 | 0.726 | 0.146 | 3.404 | **0.001** |
|  | 3 | 0.570 | 0.170 | 2.416 | **0.019** |  |  | 3 | 0.581 | 0.127 | 6.871 | **0.000** |
|  | insignificant | 0.662 | 0.141 | -0.398 | 0.691 |  |  | insignificant | 0.727 | 0.154 | 3.651 | **0.000** |
| MinMeanE | control | 0.610 | 0.104 |  |  |  | MinMeanD | control | 0.762 | 0.078 |  |  |
|  | 1 | 0.594 | 0.191 | 0.462 | 0.646 |  |  | 1 | 0.692 | 0.185 | 2.213 | **0.031** |
|  | 2 | 0.592 | 0.156 | 0.764 | 0.447 |  |  | 2 | 0.684 | 0.159 | 3.754 | **0.000** |
|  | 3 | 0.394 | 0.175 | 5.081 | **0.000** |  |  | 3 | 0.405 | 0.152 | 9.875 | **0.000** |
|  | insignificant | 0.593 | 0.167 | 0.779 | 0.438 |  |  | insignificant | 0.686 | 0.167 | 3.946 | **0.000** |
| MinWash | control | 11.910 | 7.516 |  |  |  | MaxWash | control | 22.401 | 9.542 |  |  |
|  | 1 | -4.218 | 29.178 | 3.425 | **0.001** |  |  | 1 | 16.536 | 31.695 | 1.133 | 0.263 |
|  | 2 | -3.230 | 27.173 | 4.882 | **0.000** |  |  | 2 | 15.393 | 26.664 | 2.197 | **0.030** |
|  | 3 | -26.26 | 50.098 | 3.389 | **0.003** |  |  | 3 | 19.266 | 35.530 | 0.388 | 0.702 |
|  | insignificant | -3.539 | 27.707 | 5.729 | **0.000** |  |  | insignificant | 15.751 | 28.216 | 2.301 | **0.023** |
