# Supplementary material for: Semiquantitative assessment of 99mTc-MIBI uptake in parathyroids of secondary hyperparathyroidism patients with chronic renal failure
Source: Front Endocrinol (Lausanne). 2022 Sep 8;13:915279. doi: 10.3389/fendo.2022.915279 (PMC9492857; doi:10.3389/fendo.2022.915279)
Supplement: Supplementary file 8 [file Table_5.docx]

**Supplementary Table 5** The correlations of both indices in CRF patients

| indices | items | age | course | hemodialysis months | GFR | AKP | BUN | CRE | UA | BUN/Cre |
| --- | --- | --- | --- | --- | --- | --- | --- | --- | --- | --- |
| Hb  (g/l) | *r* | 0.038 | **0.267^b^** | **0.320^c^** | **0.401^a^** | 0.112 | **-0.253^b^** | -0.003 | 0.034 | -0.147 |
|  | cases | 151 | 151 | 151 | 37 | 145 | 150 | 150 | 150 | 150 |
| age  (years) | *r* |  | **0.305^c^** | **0.197^a^** | -0.144 | -0.147 | -0.146 | **-0.232^b^** | **-0.166^a^** | -0.034 |
|  | cases |  | 151 | 151 | 37 | 145 | 150 | 150 | 150 | 150 |
| Course  (months) | *r* |  |  | **0.709^c^** | -0.247 | **0.208^a^** | -0.095 | 0.066 | -0.160^a^ | **-0.211^b^** |
|  | cases |  |  | 151 | 37 | 145 | 150 | 150 | 150 | 150 |
| hemodialysis months | *r* |  |  |  | **-0.336^a^** | **0.283^c^** | **-0.188^a^** | 0.129 | **-0.180^a^** | **-0.342^c^** |
|  | cases |  |  |  | 37 | 145 | 150 | 150 | 150 | 150 |
| GFR (ml/min) | *r* |  |  |  |  | -0.300 | **-0.376^a^** | **-0.392^a^** | -0.050 | 0.288 |
|  | cases |  |  |  |  | 35 | 37 | 37 | 37 | 37 |
| AKP  (U/L) | *r* |  |  |  |  |  | -0.101 | -0.021 | -0.115 | -0.103 |
|  | cases |  |  |  |  |  | 145 | 145 | 145 | 145 |
| BUN  (mmol/L) | *r* |  |  |  |  |  |  | **0.535^c^** | **0.396^c^** | **0.428^c^** |
|  | cases |  |  |  |  |  |  | 150 | 150 | 150 |
| Creatinine (μmol/L) | *r* |  |  |  |  |  |  |  | **0.205^a^** | **-0.426^c^** |
|  | cases |  |  |  |  |  |  |  | 150 | 150 |
| UA  (μmol/L) | *r* |  |  |  |  |  |  |  |  | **0.326^c^** |
|  | cases |  |  |  |  |  |  |  |  | 150 |

**Supplementary Table 5** The correlations of both indices in CRF patients – to be continued

| indices | items | Ca | P | Ca × P | CPI | calcitonine | PTH | Ferritin | VitB12 | folate | EPO |
| --- | --- | --- | --- | --- | --- | --- | --- | --- | --- | --- | --- |
| Hb  (g/l) | *r* | **0.389^c^** | 0.008 | **0.237^b^** | **0.262^b^** | 0.095 | -0.045 | 0.050 | 0.152 | **0.179^a^** | -0.006 |
|  | cases | 151 | 151 | 151 | 145 | 151 | 151 | 151 | 151 | 151 | 144 |
| age  (years) | *r* | -0.120 | **-0.271^c^** | **-0.291^c^** | -0.058 | **-0.199^a^** | -0.140 | -0.046 | 0.126 | -0.018 | 0.103 |
|  | cases | 151 | 151 | 151 | 145 | 151 | 151 | 151 | 151 | 151 | 144 |
| Course  (months) | *r* | **0.212^b^** | -0.005 | 0.125 | **0.452^c^** | -0.024 | **0.177^a^** | 0.105 | **0.271^c^** | **0.212^b^** | 0.124 |
|  | cases | 151 | 151 | 151 | 145 | 151 | 151 | 151 | 151 | 151 | 144 |
| hemodialysis months | *r* | **0.288^c^** | -0.043 | 0.135 | **0.566^c^** | 0.033 | **0.226^b^** | 0.125 | **0.309^c^** | **0.272^c^** | **0.201^a^** |
|  | cases | 151 | 151 | 151 | 145 | 151 | 151 | 151 | 151 | 151 | 144 |
| GFR (ml/min) | *r* | 0.224 | -0.298 | -0.077 | **-0.601^c^** | -0.109 | **-0.456^b^** | -0.269 | -0.078 | 0.191 | -0.215 |
|  | cases | 37 | 37 | 37 | 36 | 37 | 37 | 37 | 37 | 37 | 36 |
| AKP  (U/L) | *r* | 0.024 | -0.056 | -0.004 | **0.175^a^** | -0.030 | **0.588^c^** | **0.280^c^** | 0.087 | -0.102 | 0.107 |
|  | cases | 145 | 145 | 145 | 143 | 145 | 145 | 145 | 145 | 145 | 138 |
| BUN  (mmol/L) | *r* | **0.287^c^** | **0.564^c^** | **0.316^c^** | 0.010 | 0.097 | 0.032 | 0.041 | -0.005 | 0.008 | -0.155 |
|  | cases | 150 | 150 | 150 | 145 | 150 | 150 | 150 | 150 | 150 | 143 |
| Creatinine (μmol/L) | *r* | -0.105 | **0.632^c^** | **0.471^c^** | **0.439^c^** | **0.239^b^** | 0.084 | 0.092 | -0.042 | 0.085 | -0.105 |
|  | cases | 150 | 150 | 150 | 145 | 150 | 150 | 150 | 150 | 150 | 143 |
| UA  (μmol/L) | *r* | -0.036 | **0.255^b^** | **0.194^a^** | -0.092 | 0.042 | -0.002 | 0.062 | -0.036 | -0.011 | **-0.202^a^** |
|  | cases | 150 | 150 | 150 | 145 | 150 | 150 | 150 | 150 | 150 | 143 |
| BUN/Cre | *r* | -0.120 | -0.066 | -0.118 | **-0.456^c^** | -0.103 | -0.083 | -0.073 | -0.029 | -0.029 | -0.059 |
|  | cases | 150 | 150 | 150 | 145 | 150 | 150 | 150 | 150 | 150 | 143 |
| Ca  (mmol/L) | *r* |  | -0.075 | **0.476^c^** | **0.253^b^** | 0.158 | 0.014 | 0.042 | 0.024 | 0.146 | 0.020 |
|  | cases |  | 151 | 151 | 145 | 151 | 151 | 151 | 0.151 | 151 | 144 |

**Supplementary Table 5** The correlations of both indices in CRF patients – to be continued

| indices | items | Ca | P | Ca × P | CPI | calcitonine | PTH | Ferritin | VitB12 | folate | EPO |
| --- | --- | --- | --- | --- | --- | --- | --- | --- | --- | --- | --- |
| phosphorus  (nmol/L) | *r* |  |  | **0.828^c^** | **0.317^c^** | **0.182^a^** | **0.213^b^** | 0.029 | 0.070 | 0.100 | -0.124 |
|  | cases |  |  | 151 | 145 | 151 | 151 | 151 | 151 | 151 | 144 |
| Ca × P | *r* |  |  |  | **0.443^c^** | **0.246^b^** | **0.220^b^** | 0.061 | 0.086 | **0.175^a^** | -0.094 |
|  | cases |  |  |  | 145 | 151 | 151 | 151 | 151 | 151 | 144 |
| CPI  (mg/L) | *r* |  |  |  |  | **0.183^a^** | **0.260^b^** | **0.198^a^** | 0.063 | 0.136 | **0.198^a^** |
|  | cases |  |  |  |  | 145 | 145 | 145 | 145 | 145 | 139 |
| calcitonine (pg/mL) | *r* |  |  |  |  |  | -0.052 | **0.241^b^** | -0.070 | -0.036 | 0.041 |
|  | cases |  |  |  |  |  | 151 | 151 | 151 | 151 | 144 |
| PTH  (pg/mL) | *r* |  |  |  |  |  |  | **0.197^a^** | 0.144 | -0.091 | 0.083 |
|  | cases |  |  |  |  |  |  | 151 | 151 | 151 | 144 |
| Ferritin  (ng/mL) | *r* |  |  |  |  |  |  |  | **0.195^a^** | -0.152 | 0.001 |
|  | cases |  |  |  |  |  |  |  | 151 | 151 | 144 |
| VitB12 (pmol/L) | *r* |  |  |  |  |  |  |  |  | **0.188^a^** | **0.175^a^** |
|  | cases |  |  |  |  |  |  |  |  | 151 | 144 |
| Folate  (nmol/L) | *r* |  |  |  |  |  |  |  |  |  | 0.059 |
|  | cases |  |  |  |  |  |  |  |  |  | 144 |
